# Facial feature representations in visual working memory: A reverse correlation study

**DOI:** 10.1167/jov.25.12.23

**Published:** 2025-10-24

**Authors:** Crista Kuuramo, Ilmari Kurki

**Affiliations:** 1 Department of Psychology, Faculty of Medicine, University of Helsinki, Helsinki, Finland

**Keywords:** face memory, visual working memory, psychophysical reverse correlation, psychophysics, ideal observer, forgetting, internal noise

## Abstract

For humans, storing facial identities in visual working memory (VWM) is crucial. Despite vast research on VWM, it is not well known how face identity and physical features (e.g., eyes) are encoded in VWM representations. Moreover, while it is widely assumed that VWM face representations encode efficiently the subtle individual differences in facial features, this assumption has been difficult to investigate directly. Finally, it is not known how facial representations are forgotten. Some facial features could be more susceptible to forgetting than others, or conversely, all features could decay randomly. Here, we use a novel application of psychophysical reverse correlation, enabling us to estimate how various facial features are weighted in VWM representations, how statistically efficient these representations are, and how representations decay with time. We employed the same–different task with two retention times (1 s and 4 s) with morphed face stimuli, enabling us to control the appearance of each facial feature independently. We found that only a few features, most prominently the eyes, had high weighting, suggesting face VWM representations are based on storing a few key features. A classifier using stimulus information near-optimally showed markedly similar weightings to human participants—albeit weighing eyes less and other features more—suggesting that human VWM face representations are surprisingly close to statistically optimal encoding. There was no difference in weightings between retention times; instead, internal noise increased, suggesting that forgetting in face VWM works as a random process rather than as a change in remembered facial features.

## Introduction

Accurate short-term retention of face identity representations is a crucial skill in everyday situations—imagine if you forgot the face of every new acquaintance as soon as you looked away. However, remembering facial identities is a computationally difficult problem for the brain, as individual differences in facial features are subtle, and reliable face identification requires integration of several facial features. This means that remembering and discriminating between various facial identities requires that facial representations in visual working memory (VWM), a brief and limited storage for visual information (Baddeley, 1986; Baddeley, 1992; Bays, Schneegans, Ma, & Brady, 2024; Brady, Konkle, & Alvarez, 2011; Cowan, 2001; Luck & Vogel, 2013; Ma, Husain, & Bays, 2014), must be rich yet efficient. The following question arises: How are the various physical facial features that individualize a face encoded and stored in VWM representations?

To our knowledge, no study has directly investigated how various facial features (e.g., nose, eyes, and mouth) are represented in VWM. VWM of faces has been evaluated around many themes (for review, see Gambarota & Sessa, 2019), such as familiar and unfamiliar face memory (Jackson & Raymond, 2008), effect of stimuli depicting negative emotions in recognition accuracy (Jackson, Linden, & Raymond, 2012; Jackson, Linden, & Raymond, 2013; Jackson, Wu, Linden, & Raymond, 2009; Thomas, Jackson, & Raymond, 2014), holistic face processing (Cheung & Gauthier, 2010), and psychopathology affecting face VWM (Chen, Norton, McBain, Ongur, & Heckers, 2009; Linden, Jackson, Subramanian, Healy, & Linden, 2011; Liu et al., 2021; She et al., 2019; Silver, Feldman, Bilker, & Gur, 2003; Takahashi, Hirano, & Gyoba, 2015; Yao, Chen, & Qian, 2018; Yoo et al., 2005). More generally, studies on VWM representations have explored the units of VWM storage, with researchers presenting both feature-based (Bays, 2015; Bays, Wu, & Husain, 2011; Bays & Husain, 2008) and object-based accounts (Luck & Vogel, 1997; Zhang & Luck, 2008), also using face stimuli (Jiang, Shim, & Makovski, 2008; Ölander, Muukkonen, Saarela, & Salmela, 2019).

In contrast, research on *perceptual* face representations has successfully applied psychophysical techniques to study face representations in recognition tasks. One particularly promising approach is psychophysical reverse correlation (Ahumada, 2002; Ahumada & Lovell, 1970; Murray, 2011; Neri & Levi, 2006). This method identifies the stimulus features that the visual system relies on for perceptual decisions by adding random external variation (noise) to stimulus values on a trial-by-trial basis in a discrimination task. A regression-like analysis is then used to quantify how variations in different stimulus parts/features correlate with task responses. The resulting regression weights reveal the extent to which each stimulus part/feature influences the participant's decisions.

Reverse correlation studies in face perception have shown that the eyes and mouth are especially important features for face identification. This has been demonstrated in identity discrimination with classification images (Ahumada, 2002; Ahumada & Lovell, 1970; Murray, 2011; Neri, 2018), a reverse correlation method where pixel noise is added to stimuli and the weight of each pixel is mapped spatially in the stimulus space—in a “classification image” (Creighton, Bennett, & Sekuler, 2019; Mangini & Biederman, 2004; Nagai et al., 2013; Sekuler, Gaspar, Gold, & Bennett, 2004). Another well-known variant of reverse correlation is the Bubbles method (Gosselin & Schyns, 2001), where stimuli are shown through Gaussian windows, “bubbles,” at different spatial scales. Research using this method supports the idea that the mouth and eyes are most important in face identity recognition (Royer et al., 2018; Schyns, Bonnar, & Gosselin, 2002; Vinette, Gosselin, & Schyns, 2004).

In this study, we apply reverse correlation for the first time to assess how facial features are weighted in VWM representations. Thus, we employ a novel reverse correlation method introduced by Kuuramo, Saarinen, and Kurki (2022), which can be used in a same–different change detection task commonly seen in VWM studies (Luck & Vogel, 1997; for review, see Rensink, 2002).

### Psychophysical reverse correlation in VWM

Our psychophysical reverse correlation method is conceptually very similar to previous reverse correlation methods. The aim is to find the visual stimulus features that the participant has stored in VWM representations.

Our method differs from previous studies in that we use image morphing to introduce variation in facial features instead of, for example, pixel noise or stimulus windowing. Morphing is a procedure where two face images are meshed using a computational algorithm resulting in a transformed face between the images. It has been widely applied in studies using faces as stimuli (Jiang et al., 2008; Ölander et al., 2019; Walker & Tanaka, 2003), and it yields natural-appearing face images with high ecological validity. We argue that it is an efficient way of modifying the perceptually relevant facial features compared with pixel noise because pixel noise or stimulus windowing produces only small and local changes to the image that do not often meaningfully alter the perceptual appearance of the face, requiring more trials to tease out how noise affects responses.

For our purposes, it is critical that we can manipulate each facial feature separately, as this allows us to determine how much each facial feature was used in the face identity memory task. Thus, we constructed our stimuli by morphing two faces from a set of several face identities but such that each facial feature was morphed *independently* in a continuum. This way, each feature in the resulting morphed face can resemble either one of the original faces, A or B, to a varying degree, which was determined by the feature-specific value, here called the *morph value*. The morph value ranges from 0% (feature from Identity A) to 100% (feature from Identity B). In the experimental task, participants are shown two morphed facial stimuli (the *memory* and *test stimulus*) with a brief delay in between, and they are then asked to respond whether the two faces have the same facial identity. We had a total of 40 facial identities, and the two identities that were used for the morphs on every trial were chosen at random. By using a large set of facial identities, we could ensure that participants do not learn distinctive but potentially idiosyncratic image features in face *images* but must encode and store facial *identity*.

Next, we need to determine how varying morph values for different facial features affect participants’ responses. To achieve this goal, we use linear regression with a nonlinear link function (i.e., a generalized linear model; see Knoblauch & Maloney, 2008; Knoblauch & Maloney, 2012; Murray, 2011; Pritchett & Murray, 2015) to estimate how strongly the *differences* in randomized morph values between memory and test stimulus predict a participant's responses in the task over many trials. The result of this analysis was a weight coefficient, a *memory weight*, for every facial feature that signifies the extent to which the feature was used in VWM representations. As an intuitive example of the logic of reverse correlation analysis, let us assume that the stimuli consist of just two features, eyes and mouth, and that the participant only uses the mouth as the basis of their response. Throughout the experiment, the participant should thus respond “same” if and only if the morph values in the mouth are similar in the memory and test stimuli, and “different” if the morph value difference between stimuli is larger than the participant's internal criteria for change in identity. The morph value in the eyes would not influence responses, because the eyes would not have a contribution in VWM representation. Thus, we would see a large memory weight for the mouth but not for the eyes.

### Efficiency of VWM representations studied by an optimal stimulus information classifier

We use a method similar to ideal observer analysis (Geisler, 2003; Geisler, 2011) to determine the statistical efficiency of the estimated face feature memory weights. The basic idea in ideal observer analysis is to compare the human feature weighting with a (Bayesian) model where weights are optimized such that they will maximize the identification performance. Different facial features may vary in how much information they contain, explaining why some features are weighed more than others. Features that have high physical contrast and large variation among individuals are the most informative and should thus be weighed most in identity discrimination in a statistical sense. Contrarily, low-contrast features that have little individual variation are poor for identifying the individual and should be weighted less. The ideal observer provides thus a norm of the feature weighting based on available stimulus information. Comparison with an ideal observer can therefore also reveal specific inefficiencies in human VWM, such as overrelying on the most informative features, which could result from a neural heuristic.

Our model, which we call an optimal stimulus information classifier, is image based and slightly more complex than ideal observers in many previous psychophysical studies, as we use facial morphing and several target face identities. We started by generating a large number of morph stimuli in a similar manner as in the experiment, but with a smaller difference in morph value range between the same and different trials to make the overall performance comparable to humans. We then used principal component analysis (PCA) to reduce the dimensionality of the stimulus space. PCA will find a set of orthogonal vectors (eigenfaces) that explain most of the variance in the morphed faces data set, in a similar way as in eigenface image recognition algorithm (Kirby & Sirovich, 1990; Sirovich & Kirby, 1987; Turk & Pentland, 1991). We then use Fisher's linear discriminant analysis (LDA; Duda, Hart, & Stork, 2001; see also Dhingra & Smith, 2004), which finds an optimal weighting for maximal separation of face identities in the data. As we combined both PCA and LDA, our optimal stimulus information classifier works similarly to the *Fisher faces* face recognition algorithm (Belhumeur, Hespanha, & Kriegman, 1997). We then estimated the optimal stimulus information classifier's memory weights in the morph space by estimating the LDA response (classified as “same” or “different”) for the morphed face data set and then reverse correlating this response with morph values that were used to generate the morphs, in a similar manner as in the participant data analysis. The optimal stimulus information classifier here finds the approximately optimal weighting of face image pixels such that the likelihood of making a correct discrimination between the same and different face is maximal.

Previous studies have shown that many aspects of face processing, such as the point of first fixation (Peterson & Eckstein, 2012), are in fact close to an ideal observer prediction and thus statistically efficient. Ideal observers have been used also in several other face perception studies, such as in studying facial symmetry perception (Bittner & Gold, 2017), spatial frequencies used in face perception (Gold, Bennett, & Sekuler, 1999), the face inversion effect (Sekuler et al., 2004), and facial emotion recognition (Ziebell, Collin, Rainville, Mazalu, & Weippert, 2020). However, these methods have not previously been used for analyzing the efficiency of VWM representations.

### Forgetting in VWM

In addition to feature encoding and the efficiency of face representation, a crucial yet somewhat disregarded question in VWM has been how memory representations are forgotten. VWM is known to be limited in duration (Barrouillet, De Paepe, & Langerock, 2012; Krill, Avidan, & Pertzov, 2018; Mercer & Barker, 2020; Pertzov, Manohar., & Husain, 2017; Rademaker, Park, Sack, & Tong, 2018), with forgetting occurring as fast as in a few seconds (Cohen-Dallal, Fradkin, & Pertzov, 2018; Kuuramo et al., 2022; Mercer & Barker, 2020). Different mechanisms of forgetting in VWM have been proposed such as interference from other items in memory (Lewis-Peacock & Norman, 2014; Lin & Oberauer, 2022; Mercer, 2014; Oberauer & Lin, 2017), “drift” in neural representations (Lim, Ward, Vickery, & Johnson, 2019; Wimmer, Nykamp, Constantinidis, & Compte, 2014; Wolff, Jochim, Akyürek, Buschman, & Stokes, 2020), systematic changes in VWM items (Gold, Murray, Sekuler, Bennett, & Sekuler, 2005), or simply temporal decay (Barrouillet et al., 2012; Barrouillet, Uittenhove, Lucidi, & Langerock, 2018; Mercer & Barker, 2020; Nilsson, 2020; Portrat, Barrouillet, & Camos, 2008; Rademaker et al., 2018; Ricker, Sandry, Vergauwe, & Cowan, 2020; Schneegans & Bays, 2018), possibly because of accumulation of internal noise in representations (Kuuramo et al., 2022). In other words, more research on this topic is needed.

Reverse correlation methodology allows us to characterize how face representations change over retention time (i.e., forgetting in VWM). We compare how facial feature weighting changes when time passes by comparing weightings in short and long retention time. In this way, we can directly identify these features in which the weightings change. Additionally, we ask whether forgetting in VWM is a fundamentally random process, such as the accumulation of internal noise. This would lead to random decay of features—in other words, the memory weights of face features would all decay in the same manner so that there is no change in the relative memory weights. Conversely, forgetting could erase only some memory weights, effectively making the representations more inefficient in storing the face identity, decreasing the sampling efficiency.

First, we can explore the question of random forgetting by comparing the memory weights in a short and long retention time and studying whether the weightings of different facial features change. Second, we can directly measure the internal noise in the memory representations at various time points, using the double-pass procedure (Burgess & Colborne, 1988; Green, 1964). In this method, each identical stimulus pair is presented twice, and the internal noise level is estimated from the consistency of responses. If we see an increase in the internal level with longer retention times, this indicates that the VWM representations become noisier with time and, thus, decay randomly. Third, we can measure changes in sampling efficiency by comparing the participants’ performance to our optimal stimulus information classifier model at different times. Decreasing sampling efficiency would suggest that the participant's memory weight changes and some features have been removed from a VWM representation.

A recent study by Kuuramo et al. (2022) found that internal noise increased, but VWM weighting did not change with longer retention times in a VWM task with simple spatial and radial frequency patterns. This provides support for the random decay model where forgetting is caused by the increase of internal noise in the VWM representations (i.e., the contents of the representations do not change but become noisier over time).

### Research questions

The aim of this study is to investigate what facial features are stored in VWM, the efficiency of face VWM representations, and how these representations are forgotten using a novel psychophysical reverse correlation methodology. Our specific research questions are as follows: (a) To what extent are different facial features, such as eyes and mouth, used in VWM representations of facial identity? (b) How efficient are human face representations compared with an optimal stimulus information classifier that can maximally use the available information presented? (c) How do VWM face representations change over time, and are facial features forgotten randomly because of the accumulation of internal noise?

## Methods and materials

### Participants

A total of 20 participants (8 men, aged 19–47 years, mean age 26.0 years) participated in this study. All participants reported having a normal or corrected-to-normal vision, and they gave their written consent to participate. All participants reported having normal face perception abilities. In addition, they filled out the PI20 questionnaire, which is designed to identify developmental prosopagnosia (Shah, Gaule, Sowden, Bird, & Cook, 2015). The responses to the questionnaire did not indicate abnormal face perception abilities in any participant. This study was approved by the University of Helsinki Research Ethics Committee in the Humanities and Social and Behavioural Sciences. The experiments were conducted in accordance with the guidelines of the Declaration of Helsinki.

### Equipment

The measurements were conducted in a dimmed vision research laboratory. We presented stimuli using a Mitsubishi Diamond Pro CRT monitor with a refresh rate of 100 Hz and a resolution of 800 × 600 pixels at a size of 36 × 29 cm (10.29° and 8.29° in visual angle). The mean luminance of the monitor was 48 cd/m^2^, and the maximum luminance was 96 cd/m^2^. The viewing distance (110 cm) was controlled with a chin rest. We used a Cambridge Research Systems ViSaGe Mark II graphics card with a grayscale color depth of 15 bits. The experiments were run using MATLAB (MathWorks, Natick, MA, USA) Version R2013a with the Psychophysics Toolbox extension Version 3.0.11 (Kleiner, Brainard, & Pelli, 2007). Statistical analyses were conducted using MATLAB R2018b and JASP Version 0.16.4.0.

### Stimuli

**Figure 1.**
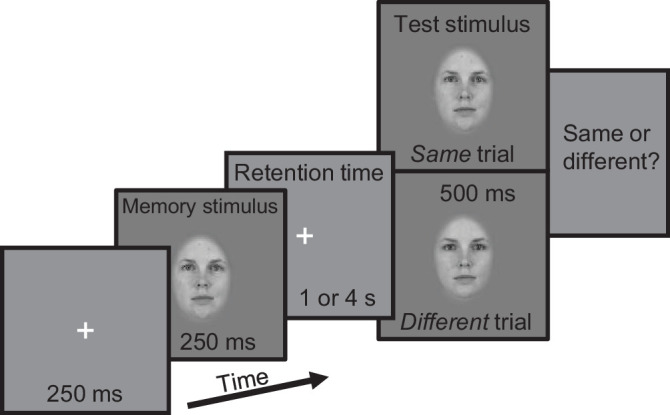
The experimental procedure. We employed a same–different change detection task, where a fixation cross (250 ms) was followed by a memory stimulus for 250 ms. After a retention time (randomly 1 or 4 s), a test stimulus was shown for 500 ms. Participants were asked to respond whether the stimuli were of the same or different identity. The stimuli were constructed such that each facial feature was morphed independently by a random amount between two identities. The amount of morphing depended on the trial type; in same trials, morph values in both stimuli were sampled from a uniform distribution of [0%, 20%]. In different trials, one of the stimuli was sampled from a uniform distribution of [0%, 20%] and the other from a uniform distribution of [0%, 80%]. Note that even though the figure depicts that the stimuli sampled from the [0%, 80%] distribution is the test stimulus, it was the memory stimulus randomly in 50% of the trials.

The stimuli were constructed from 40 photographs of Caucasian individuals (20 male) with neutral expressions selected from the Chicago Face Database version 2.0.3 (Ma, Correll, & Wittenbrink, 2015; see examples of stimuli in Figure 1). In choosing the images, we first excluded faces using the validation data provided with the data set if their average rating for angry, happy, sad, afraid, surprised, or disgusted expression had a score of 2 or more on a Likert scale from 1 to 5. We also excluded faces whose sex or ethnicity was rated erroneously over 5% of the time. After initial pruning, we also excluded faces that had visible scarring, large moles or birthmarks, or a large amount of makeup or hair covering the forehead or other parts of the face. The estimated age of the persons in the face images was not controlled; the average estimated age calculated from the validation data was 29.34 years for male faces and 26.10 years for female faces. We added a small amount of white pixel noise (RMS contrast 2.56) to the final stimuli to prevent participants from using small pixel differences between stimuli as a memory cue.

#### Morphing procedure

Before facial morphing, any hair possibly obscuring the face in the images was manually erased using the GIMP 2 image editor. To construct the face morphs, we first landmarked 140 morph points over every face image with a custom MATLAB function (see details on the morph points in Supplementary Appendix I). The points were evenly distributed over the faces such that they tracked the outlines of important facial features (e.g., line of the jaw, eyebrows, and outlines of the eyes). Some of the points were placed on cephalometric landmarks, such as the nasion, to aid placing the points in corresponding locations on each image. The morph points were grouped into seven semantically significant facial features (“morph groups” that consisted of eyes, nose, mouth, cheeks, forehead, jaw, and eyebrows; see the morph groups in Supplementary Appendix I). There were also additional morph points in the ears and the neck to smooth the edges of the face, but these areas were not considered in further analysis.

**Figure 2.**
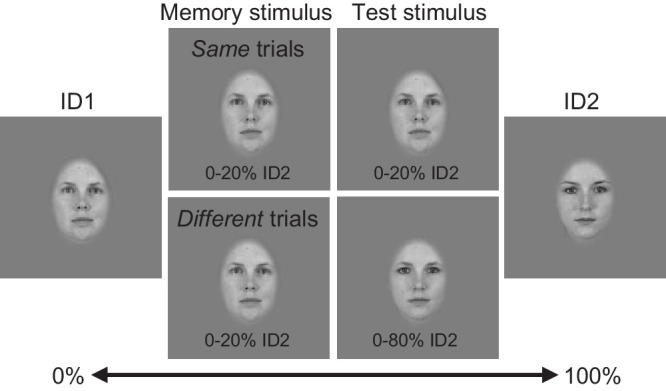
The morphing procedure. We formed a morph space between two identities at a time (ID1 and ID2), where each feature was morphed independently according to a morph value between 0% and 100% toward ID2, depending on the trial type. In same trials, both stimuli were “same” stimuli morphed in the range of [0%, 20%]. In different trials, one of the stimuli was a “different” stimulus morphed in the range of [0%, 80%] instead.

We then randomized the amount of morphing (the morph value) for each morph group separately, such that the points in one group were always morphed the same amount. The morph values could theoretically range from 0% (no morphing, corresponds to Identity 1) to 100% (morphed all the way to Identity 2), but the range of morphing depended on the trial type; in the same trials, all morphs were between 0% and 20% from Identity 1 to Identity 2 (the “same” stimulus). In different trials, the range of one of the stimuli was 0% to 80% (a “different” stimulus), while the other stimulus was the “same” stimulus (see Figure 2 for illustration). The resulting stimuli were a hybrid face where each facial feature was morphed a random amount from a base identity to a second identity. Note that all face images were morphed, even in the “same” trials where morph values were very close to Identity 1 (ID1). This was done to ensure that the possible presence or absence of cues caused by the morphing operation (e.g., changes in skin texture) could not be used as a cue. Please note that the morph ranges in “same” and “different” trials overlap, and in some “same” trials, a stimulus could be morphed more toward a second identity than in some “different” trials. This was done intentionally to make the task sufficiently difficult, as estimating a reverse correlation model requires that there are errors in responses and works best when there is a high level of external variation in the stimuli. No morphs were done between the same identity, and female/male faces were only morphed to other female/male faces, respectively, resulting in 760 (2 × 19 × 20) possible unique morphs between faces (e.g., ID1 to ID2, ID1 to ID3, etc.). We generated two versions of “different” stimuli and four versions of “same” stimuli (because only a third of stimuli were “different” stimuli), resulting in a total of 6 × 760 = 4,560 unique morphed stimuli.

#### Image processing

The morphed images were windowed with an oval Gaussian mask extending 305 × 450 pixels with a standard deviation of 76 pixels, fitted to the faces, showing only the face, excluding hair and neck (see Figure 1). The images were then subsampled to 500 × 500 pixels and transformed to grayscale with a background of middle gray. The visible portion of the face extended 8.07 × 11.90 cm and 4.20° × 6.20° in visual angle on the monitor. We chose the size of the stimuli based on Yang, Shafai, and Oruc (2014), who found that only face stimuli exceeding 6° showed an inversion effect and, thus, supposedly were processed through a specialized face recognition system.

### Procedure

We used a same–different change detection task, where the trial started with a fixation cross for 250 ms, which was followed by a memory stimulus for 250 ms. A retention time of 1,000 or 4,000 ms followed, during which a fixation cross was present. Retention time duration was randomized trial-by-trial such that each block had an equal number of both retention time durations. Then a test stimulus was shown for 500 ms. The participant's task was to indicate with a button press whether the memory and test stimuli were the same or different identities. Participants gave their answers on a 4-point rating scale: “definitely same,” “probably same,” “probably different,” or “definitely different.” Immediately after that, they received auditory feedback as a beep after a wrong answer. Response time was not limited, and the next trial began immediately after an answer was given. Half of the trials were “same” trials, where both stimuli were the “same” stimuli that on average looked like the same identity. The rest of the trials were “different” trials, where one stimulus was a “different” and the other a “same” stimulus, which made the two stimuli appear as different identities on average. The order of the “same” and “different” stimuli was randomized in “different” trials. The face identities and morph instances for each identity were also randomized. Each unique trial was repeated in a random order within each block for internal noise-level estimation (see details below). Participants conducted a total of 2,880 trials spread across five approximately hour-long sessions with seven to eight blocks in each session. A single block contained 80 trials. Before the measurements, a short practice block of approximately 20 trials was run that was not included in the analysis.

### Data analysis

#### Performance

Performance in the same–different task was estimated with *d*′, a signal detection theory measure that measures how accurately the participant can discriminate between the same and different stimuli.

#### Reverse correlation analysis

Memory weights were estimated using a generalized linear model (GzLM; see Knoblauch & Maloney, 2008; Knoblauch & Maloney, 2012; Murray, 2011; Pritchett & Murray, 2015). We used the same estimation procedure in the reverse correlation analysis and internal noise estimation as in Kuuramo et al. (2022).

We assume that a participant makes a same/different decision by comparing the difference between memory and test stimuli on a set of stimulus features. The aim of the analysis is to estimate the memory weights that the participant has put on different features when making the same/different decisions. We denote these weights by the vector **w**. The memory weight vector **w** is estimated as follows: Let us assume a participant makes a decision using the decision variable *r_k_* in every trial *k*. The decision variable calculates a weighted sum of the memory weights and the squared value of the difference between morph values in memory and the test stimulus, denoted by **n**:

(1) $$\begin{array}{c} r_{k}=w^{T}n_{k}^{2}+{ɛ}_{k},\; \end{array}$$

where ε_*k*_ is Gaussian and independent random internal noise in trial *k*. Note that the task was to decide if two presented faces were the same or different. Therefore, we assume that the observer is sensitive to the difference between memory and retention morph values, regardless of the identity axis direction, and use squared values. Note further that the morph value distribution here is uniform. However, the distribution of the decision variable can be assumed to be approximately Gaussian according to the central limit theorem as the number of features involved in comparison is large. We further assume that the participant has an internal confidence rating by comparing the decision variable *r_k_* to a set of internal criteria. Thus, the participant gives a rating of *j* when the decision variable is between criteria *c_j_* and *c*_*j* + 1_. If internal noise is Gaussian, the expected value of the probability of a difference response with confidence *l* or greater can be written as

(2) $$\begin{array}{c} E\left(p\left(r_{k}>c_{l}\right)\right)=\Phi \left(w^{T}n_{k}^{2}-c_{l}\right),\; \end{array}$$

where Φ is the standard cumulative normal distribution function.

Our GzLM model had 12 regressors for facial feature and 3 for response categories determined by the internal criteria. We used an ordinal probit link function. To estimate the model, we used MATLAB's *mnrfit* function. For comparing the memory weights across the observers, we divided the regression weights (memory weights) by their vector length to normalize them for every participant. This was done as the magnitude of the weights estimated by the GzLM is related to the internal noise (Ahumada, 2002; Knoblauch & Maloney, 2008), and we wanted to eliminate its effect on the results. As a control, we ran an analysis using only “different” trials and found essentially identical results.

#### Internal noise estimation using double-pass response inconsistency

To estimate internal noise, we used the double-pass response consistency method (Burgess & Colborne, 1988; Green, 1964). In this method, each trial is repeated twice; thus, any difference in responses must be caused by internal noise. We can estimate the relative amount contributed by internal noise by measuring the consistency of the responses, that is, the probability of giving two identical responses in repeated trials, as described in Kuuramo et al. (2022). As in our task the responses were given using a rating scale, we used a modification of the double-pass consistency procedure generalized for rating scale responses (Kurki & Eckstein, 2014). Note that the analysis assumes that the responses to both the internal noise and external variation to be approximately Gaussian. As we previously mentioned, the probability density function for *r_s_* is strictly speaking not normal, but we approximate it by a normal distribution based on the central limit theorem.

#### Optimal stimulus information classifier

**Figure 3.**
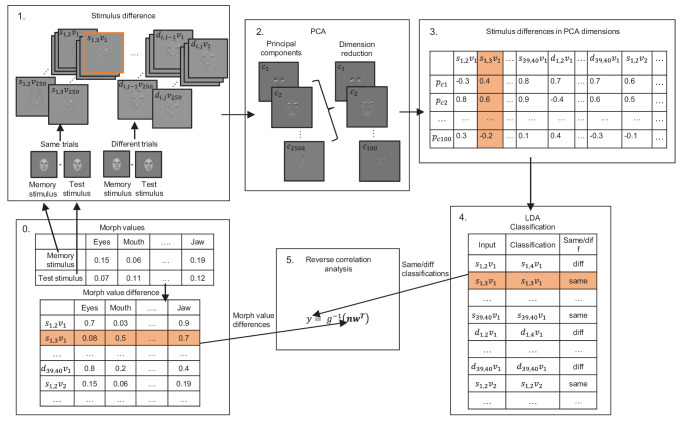
A flowchart of the optimal stimulus information classifier analysis. The aim of the analysis is to find an optimal weighting of facial features for same/different discrimination. The orange color signifies a single instance of a stimulus pair throughout the analysis pipeline. 0. For each facial feature, a morph value is randomized in both memory and test stimulus. Morph values were randomized for “same” (s) and “different” (d) stimuli using different morph value ranges. 1. A large number of face stimulus images were generated. *s*_*x*,*y*_*v_z_* denotes the *z*th randomized instance of the face stimulus (pixel) difference between “same” stimulus x and y. Respectively, *d*_*x*,*y*_*v_z_* denotes a “different” stimulus. 2, 3. Stimulus differences were first projected to a PCA space, and then the first 100 principal components c were included in the remaining analysis. 4. The LDA model was then used to classify these stimulus differences’ identity categories (same/different stimulus × face identity). 5. To find the weighting w for facial features used by the optimal stimulus information classifier, we use reverse correlation analysis. It finds the ideal weights (w) for the morph value differences between the same and different stimuli (matrix n) to predict the same/different response (y) of the LDA model.

We conducted an analysis similar to a Bayesian ideal observer to study how efficiently observers were able to extract and retain information from the face stimuli used here. The idea of the analysis is to use the same stimuli as we did in the behavioral experiment (however, with a larger number of morphed face pair instances). Then we used an optimal image-based classifier that has perfect knowledge of discriminating information (at the image level) in face identity pairs. We performed the same reverse correlation analysis on morph values used to generate the face identity pairs, now based on the outputs of the optimal classifier. Therefore, we can compare human memory weights to a model that uses the stimulus information optimally. The model, to which we refer as an optimal stimulus information classifier (see Figure 3 for illustration), is based on Fisher's LDA, which is an optimal classifier if stimulus values are statistically linearly correlated. As LDA requires estimation of the covariance matrix and is computationally demanding with larger stimulus spaces, we first reduced the dimensionality and orthogonalized the stimulus space by using PCA.

The optimal stimulus information classifier performance was approximately matched to the human level of performance at the shorter 1,000-ms retention time by using a smaller morph value range in “different” stimuli, making the task more difficult. In “same” stimuli, the morph range was identical to the experiment. It was necessary to adjust the performance of the classifier, as reverse correlation analysis cannot be performed if the classifier performance is perfect, as it would have been otherwise. We also tested different morph ranges for the classifier and found that the classifier memory weights were not highly sensitive to the exact ranges used. There were 760 × 2 unique *face pair types* (i.e., morphs between the face identities that were either “same” or “different” stimuli). For each of these face pair types, we made 250 image pairs with random morph instances. We then spatially subsampled the images (one of five pixels in both horizontal and vertical dimensions) to reduce correlations and dimensionality. To reduce the stimulus dimensionality further, we used PCA to find an orthogonal linear transformation of the original data such that new axes (principal components, or eigenvectors) are aligned to the maximum variance in the data. We then took the 100 principal components with the highest eigenvalues. We tested models with different amounts of main components but found no marked differences in the results. We used the quadratic LDA to predict the stimulus pair's face pair type using the *fitcdiscr* algorithm of MATLAB R2024a. The quadratic discriminant option was used, allowing the covariance matrices to vary across the classes. We evaluated both the accuracy for the correct face pair type identification and the same/different superclass of the identity response (regardless of face identity response). The 10-fold cross-validated accuracy of the classifier was about 71.8% for identifying the correct face pair type and about 73.6% for the same/different superclass identification. We then estimated the ideal memory weights using the GzLM in a similar manner to that of the participants. The morph values used to generate images served as regressors, while the LDA same/different responses were used as the response variable. Note that the method gives a single optimal weight vector that can be thought of as an average over the identities, even when best identifying features may vary by identity pair and is taken into account by the optimal stimulus information classifier. To measure how close the human feature weighting is to an ideal observer, we estimated sampling efficiency of human memory weights. It is defined as the dot product between the normalized human memory weight vector and the normalized ideal memory weight vector (e.g., Ahumada, 2002).

#### Statistical analysis

We compared the memory weights in different retention times and across facial feature groups by conducting a two-way repeated-measures analysis of variance (ANOVA). Multiple comparisons were accounted for using the Bonferroni correction. When specified, we used the false discovery rate (FDR) instead to account for multiple comparisons (Benjamini & Hochberg, 1995). We compared *d*′ values between retention times using a repeated-measures *t*-test. We also ran equivalent Bayesian analyses in addition to frequentist analyses.

## Results

### Task performance and internal noise

**Figure 4.**
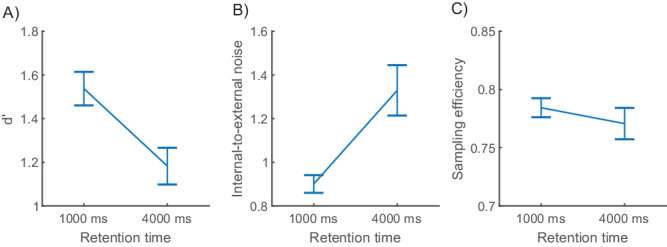
(**A**) Performance measured as *d*′. (**B**) Mean internal noise measured by the double-pass procedure. (**C**) Mean sampling efficiency. All error bars represent *SEM*.

We found an approximately 23% decrease in task performance measured as *d*′ with retention time (*M*_1__,__000_ = 1.54, *SD*_1__,__000_ = 0.33; *M*_4__,__000_ = 1.18, *SD*_4__,__000_ = 0.36; see Figure 4A). In a repeated-measures *t*-test, *d*′ decreased statistically significantly from 1,000-ms to 4,000-ms retention times: *t*(17) = 10.27, *p* < 0.001, *d* = 1.04. In an equivalent Bayesian test, we found a Bayes factor of 1.17 × 10^6^ in favor of the alternative hypothesis, providing very strong evidence for a decrease in performance with a longer memory time. Note that each stimulus pair was presented twice. If some information about the stimulus were encoded in long-term memory in the first pass, this might inflate the internal noise values, as performance in both passes is assumed to be identical in the double-pass method. We tested whether *d*′ differed between passes in a repeated-measures *t*-test and found no effect of pass on *d*′: *M*_1_ = 1.35, *SD*_1_ = 0.37; *M*_2_ = 1.38, *SD*_2_ = 0.37, *t*(17) = 0.86, *p* = 0.202, *d* = 0.12. We conducted an equivalent Bayesian test, where we found a Bayes factor of 2.51 favoring the null hypothesis, further suggesting that there was no long-term memory effect between passes on performance. We further tested whether there is a long-term memory component in *d*′ between passes on different retention times using a two-way ANOVA; we found no effect for pass (*F*(1, 16) = 0.85, *p* = 0.370, $\eta_{p}^{2}$ = 0.05) but, as expected, found an effect for retention time (*F*(1, 16) = 93.95, *p* < 0.001, $\eta_{p}^{2}$ = 0.85).

The ratio of internal to external variability, measured by the double-pass procedure, increased about 48% with retention time (*M*_1,000_ = 0.90, *SD*_1,000_ = 0.04; *M*_4,000_ = 1.33, *SD*_4,000_ = 0.12, see Figure 4B). We tested whether this difference was significant with a repeated-measures *t*-test and found a strong effect of retention time: *t*(17) = 3.75, *p* = 0.002, *d* = 1.17. In a Bayesian *t*-test, the alternative hypothesis was 24.78 times more likely than the null hypothesis, providing strong evidence for the notion that the internal noise ratio declined with retention time.

### Psychophysical reverse correlation model

**Figure 5.**
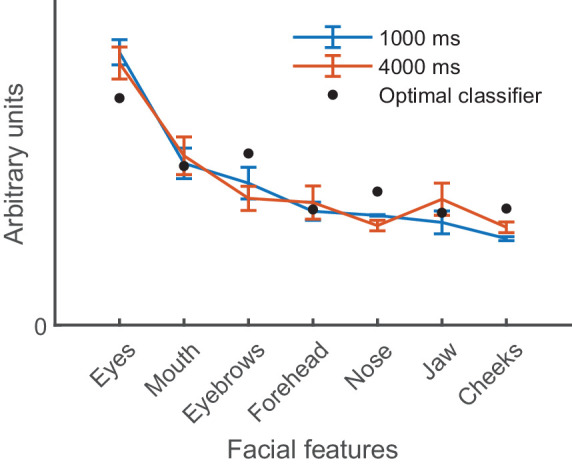
Average normalized memory weights for short (1,000 ms) and long (4,000 ms) retention time for each feature in the x-axis (lines) and average weights of a simulated optimal stimulus information classifier model (black circles). Weights are sorted from largest to smallest according to the weights of the human participants in the 1,000-ms retention time. Error bars represent *SEM*. The higher the weights, the more the feature affected participants’ responses. A weight of zero indicates that the feature had no effect on participants’ responses.

On visual inspection, we found positive memory weights for all facial features (see Figure 5 for grand average weights and Supplementary Appendix II for feature weightings for each participant separately). The eyes were the most prominent feature used in the task, with weights accounting for about 28% of all feature weights summed together in the 1,000-ms retention time and about 26% in the 4,000-ms retention time. The weights for mouth and eyebrows had the second and third largest memory weights, respectively. Other features (forehead, nose, jaw, and cheeks) had smaller but positive weightings. All weights were significantly larger than zero in both retention times in multiple FDR-corrected *t*-tests (*p* < 0.05).

The weighting is very similar between retention times in each facial feature. To test whether the normalized memory weights differ in the two retention intervals and the facial features, we conducted a two-way repeated-measures ANOVA. We found a significant main effect for feature (*F*(6, 102) = 30.18, *p* < 0.001, $\eta_{p}^{2}$ = 0.640), but there was no effect for retention time (*F*(1, 17) = 0.47, *p* = 0.505, $\eta_{p}^{2}$ = 0.027). This suggests that the weighting depended on the facial feature, but there was no evidence that memory weights differed in the two retention times; in other words, the performance decay was not reflected in the extent to which feature information was used. The interaction between feature and retention time was not significant (*F*(6, 102) = 0.63, *p* = 0.706, $\eta_{p}^{2}$ = 0.036). This implies that the feature weighting did not change with retention time. Using Bayesian repeated-measures ANOVA, the largest Bayes factor was in a model where feature is a sole predictor; this model was 2.66 × 10^17^ times more likely than the null model, supporting the notion that features varied in memory weight. The model also including retention time as a predictor was 4.96 × 10^16^ times more likely than the null model, and the model including both variables and their interaction as predictors was 3.76 × 10^15^ times more likely than the null model. The analysis did not support the model of retention time as the only predictor, as the Bayes factor was only 0.19, favoring the null model.

### Optimal stimulus information classifier

The optimal stimulus information classifier memory weights approximate the optimal feature weighting in the task. The largest weights were in the eyes, followed by the eyebrows, mouth, nose, cheeks, jaw, and forehead (see black circles in Figure 5). We found that the optimal stimulus information classifier memory weights were very similar to those of the human participants. However, there were also slight differences, with the weight in eyes being lower and feature weightings in eyebrows, nose, and cheeks being larger for the optimal stimulus information classifier than for human participants. Similarly to human participants, the weight in the eyes was a large proportion of all weights summed together (22%).

Sampling efficiency reveals how closely human weights correspond to optimal stimulus information weighting (i.e., how efficiently the available stimulus information can be used by human participants in the task). We found no effect of memory time on sampling efficiency (*M*_1,000_ = 0.78, *SD*_1,000_ = 0.03; *M*_4,000_ = 0.77, *SD*_4,000_ = 0.06; see Figure 4C) with a repeated-measures *t*-test (*t*(17) = 1.51, *p* = 0.150, *d* = 0.29). In a Bayesian repeated-measures *t*-test, the null hypothesis was 1.58 times more likely than the alternative hypothesis, providing weak evidence that sampling efficiency was not affected by retention time.

### Individual differences in VWM performance

We also saw somewhat large individual differences in overall performance in the VWM task. Could efficiency or internal noise explain individual variation in performance? We found no correlation between sampling efficiency and *d*′ (*r* = −0.14, *p* = 0.590), but we found a negative correlation between the internal-to-external variation ratio and *d*′ (*r* = −0.59, *p* = 0.001). Thus, individual differences in VWM performance seem to be related to variation in internal noise.

Can individual differences in forgetting be further explained by changes in efficiency or internal noise between retention times? We calculated the correlation between the individual differences in efficiency between 1,000-ms and 4,000-ms retention times and the difference in *d*′ between retention times and found no correlation (*r* = 0.10, *p* = 0.690). Again, we did not find any correlation between the individual difference in internal noise and in performance between retention times (*r* = −0.33, *p* = 0.176).

## Discussion

In this study, we investigated how different facial features are weighted in VWM representations of facial identity in a same–different change detection task using a novel method based on psychophysical reverse correlation. A unique feature of this method is that it can reveal the contribution of individual facial features in VWM representations of face identity by estimating memory weights for each feature. Additionally, we compared optimal stimulus information classifier memory weights with those of human participants to assess how close human feature-encoding strategies in VWM are to optimal feature weighting. Finally, we examined how facial feature information decays over time by examining how memory weights changed across two different retention times in our VWM task. Additionally, we measured internal noise and sampling efficiency in two retention times. This allowed us to test whether memory decay erases some features systematically in VWM representations seen in decreasing sampling efficiency or whether all features decay in a uniform manner caused by an increase in internal noise.

### VWM face representations revealed by psychophysical reverse correlation

From the psychophysical reverse correlation analysis, we extracted the memory weights for facial features. Overall, we found that there were large memory weights for only a subset of facial features. Eyes were the most highly weighted facial feature in VWM representations of face identity. The second and third largest weights, on average, were in the mouth and eyebrows, respectively. The rest of the features (forehead, nose, jaw, and cheeks) had, on average, positive but small weights. Therefore, our results suggest that the participants mostly relied on a few facial features when storing facial identity.

Our results regarding the eyes and the mouth as the most important facial features in VWM of facial identity are consistent with previous reverse correlation studies, which have found that eyes and sometimes also the mouth are used in discriminating between facial identities (Creighton et al., 2019; Mangini & Biederman, 2004; Nagai et al., 2013; Schyns et al., 2002; Sekuler et al., 2004; Vinette et al., 2004). Apart from reverse correlation studies, some studies have found that occluding especially the eyes but also the mouth from a face image impairs recognition ability (Graham & Ritchie, 2019; McKelvie, 1976; Or et al., 2023), supporting the notion that the eyes and the mouth contain particularly useful information in face identity processing.

Unexpectedly, the memory weight for the eyebrows was the third highest among the participants, despite eyebrows not typically being considered an especially important feature in face identity discrimination. However, Sadr, Jarudi, and Sinha (2003) proposed that eyebrows may play a larger role in face perception than previously thought, as removing eyebrows from a face stimulus impaired familiar face recognition more than removing the eyes. Eyebrows do have large contrast and variation between individuals, as do the eyes and mouth, which could promote their use in face identity representations. The optimal stimulus classifier had a high weight for eyebrows, consistent with this notion. One alternative explanation for the high weight for eyebrows is that they are near the eyes, a feature that has been consistently shown to be important in face discrimination. However, our method allows us to manipulate both the eye and eyebrow areas separately, and thus it is improbable that high weight in eyebrows resulted from proximity to the eyes. Another possibility is that there exists some perceptual process where a high contribution of eyes cascades to adjacent areas, possibly due to spatial attention directed to the eyes overlapping with eyebrows. However, in this case, we should also see high weights under the eyes (here, cheeks), which we did not observe. It might be that eyebrows are used more in face representations than thought before, and this could be more carefully addressed in future research.

Note also that we were able to study VWM representations using reverse correlation for the first time, as previous results using this method have been on perceptual representations. This means that we can also, for the first time, compare perceptual representations of faces with VWM face representations revealed by reverse correlation. Interestingly, we found that the same features (eyes and mouth) important in perceptual representations of faces (Creighton et al., 2019; Mangini & Biederman, 2004; Nagai et al., 2013; Royer et al., 2018; Schyns et al., 2002; Sekuler et al., 2004; Vinette et al., 2004) are also used in VWM representations of facial identity. Even though we cannot directly compare the set of facial features used in previous perceptual studies with the features used in face identity VWM, our results suggest that VWM representations retain qualities of perceptual representations to a large extent, as proposed in earlier research on VWM of color hue (Nilsson, 2020).

### Optimal stimulus information classifier

We used PCA- and LDA-based ideal observer analysis to estimate the statistically optimal weighting of identity information in faces. Remarkably, the optimal stimulus information classifier weighting of facial features was very similar to that of human participants. The optimal stimulus information classifier model and humans roughly shared the order and the magnitude of the most important facial features. The eyes had the largest memory weights, followed by moderately large weights in the mouth and eyebrows, and other features having smaller but positive weighting. Thus, the eye region–focused weighting of facial features seen in human VWM representations can, in fact, be close to an optimal solution for discriminating face identity. This is in line with previous ideal observer and eye movement studies on face perception, where fixations during face recognition are observed to land near the eyes on the nose region (Barton, Radcliffe, Cherkasova, Edelman, & Intriligator, 2006; Chelnokova & Laeng, 2011; Henderson, Williams, & Falk, 2005; Hsiao & Cottrell, 2008; Or et al., 2015; Peterson & Eckstein, 2012; Sekiguchi, 2011; Yarbus, 1967) or in a T-shaped area around the eyes and down the nose to the mouth (e.g., Arizpe, Walsh, Yovel, & Baker, 2017; Blais, Jack, Scheepers, Fiset, & Caldara, 2008; Malcolm, Lanyon, Fugard, & Barton, 2008; Walker-Smith, Gale, & Findlay, 1977; Yarbus, 1967). It has been suggested that this pattern of fixation is an optimal strategy for face recognition performance, as it maximizes information-gathering from informative facial areas such as the eyes (Hills, Ross, & Lewis, 2011; Or, Peterson, & Eckstein, 2015; Peterson & Eckstein, 2012).

The optimal stimulus information classifier model, however, had a smaller weight for eyes than human participants and, accordingly, larger weights for the other features. It seems that humans have slightly inefficient representations of face identity VWM, as they put too much weight on the most informative features while discounting less informative features. The high weighting for the eyes in humans could be partly explained by the role of the eyes in social processing. Eye perception might serve additional evolutionary functions in addition to identity discrimination, as eyes convey abundant information about, for example, emotional state and gaze direction, both integral to social information processing (for review on the role of eye gaze in social cognition, see Itier & Batty, 2009).

Even though humans used a slightly suboptimal sampling strategy, they were still highly efficient in extracting information from facial features, as the weighting of human participants was very close to the optimal stimulus information classifier. The sampling efficiency was rather high, around 78%, suggesting that face VWM representations are efficient, and VWM is limited by the large amount of internal noise instead of the suboptimal encoding and sampling strategy, at least when only a single face needs to be represented. Further, our results also suggest that individual differences in face VWM are better explained by differences in internal noise, rather than differences in encoding and sampling efficiency. One possible reason for this result can be that face VWM encoding (at least in healthy young adults) is highly learned through years of extensive training, leading to a sampling/encoding strategy close to optimal. However, this result may not generalize to all populations, such as prosopagnostics or super recognizers (Ramon, Bobak, & White, 2019; Russell, Duchaine, & Nakayama, 2009; Tardif et al., 2019), which could be a potential avenue for future research. Note that some previous reverse correlation studies (e.g., Creighton et al., 2019; Sekuler et al., 2004; Ziebell et al., 2020) have reported much lower sampling efficiency values presumably because they had more estimation noise.

### Mechanisms of forgetting in VWM

Finally, we asked how forgetting is reflected in the memory weights of different facial features and whether forgetting faces in VWM occurs due to internal noise accumulating in representations over time. Conversely, we asked whether the representations become more inefficient (specifically, more concentrated in some key feature(s)) when time passes. With reverse correlation, we could directly examine how VWM representations change with longer retention times. A random forgetting model predicts no change in the relative weightings of stored facial features between retention times. In contrast, if efficiency declines, we would expect changes in the relative weightings. We did not find an interaction between the feature and retention time (1,000 ms or 4,000 ms) in a repeated-measures ANOVA, implying that all facial features decayed similarly. Thus, our results support a random forgetting model where internal noise corrupts VWM representations, leading to uniform decay of memory weights.

We found more evidence for the random forgetting model of VWM by directly measuring internal noise and sampling efficiency in the two retention times. Internal noise, measured by the double-pass procedure, increased in the longer retention interval, suggesting that forgetting in VWM is driven by noisier representations with time. In addition, we observed no change in sampling efficiency, indicating that participants did not become more inefficient when storing facial information. Although no study has examined the accumulation of internal noise in face VWM, a few studies have attributed forgetting in VWM to the accumulation of internal noise (Gao & Bentin, 2011; Kuuramo et al., 2022), while one study failed to find an effect of retention time on internal noise (Gold et al., 2005). We now have evidence that the same kind of process of internal noise accumulation might be behind the forgetting of more complex stimuli, namely, faces.

Our results support random forgetting caused by the increase in internal noise, but the exact mechanism by which internal noise operates cannot be determined based on our results. A signal detection theory-based model can only measure the effects of internal, random noise and the efficiency of stimulus weighting. There are multiple ways our results can be interpreted. In recent neural network models, forgetting is modeled as neural drift or diffusion caused by internal noise, where the group of neurons maintaining the activity shifts. Tomić and Bays (2024) incorporated temporal forgetting in the neural resource model of VWM (also called the population coding model; see Bays, 2014). The neural resource model assumes that VWM representations are encoded as sustained spiking activation of a population of neurons. The revised model (dynamic neural resource model or DyNR) incorporates a temporal aspect in the neural resource model and assumes that decay in both sensory and mnemonic processes affects the fidelity of VWM representations at retrieval. The same idea of forgetting is incorporated in attractor models of VWM (Panichello, DePasquale, Pillow, & Buschman, 2019; Penny, 2024), which similarly assume a random drift in VWM representations with time. Our results support this idea of forgetting, and neural drift serves as a plausible explanation of how VWM representations decay at a neural level. However, other options could similarly explain how internal noise affects representations, such as straightforward signal decay, where internal noise effectively reduces the signal-to-noise ratio of VWM representations. This model has been implied in previous studies that have found that simply the passage of time results in forgetting in VWM (Barrouillet et al., 2012; Barrouillet et al., 2018; Barrouillet & Camos, 2012; Mercer & Barker, 2020; Nilsson, 2020; Portrat et al., 2008; Rademaker et al., 2018; Ricker et al., 2020), but it has not been explicitly tested. Further, our results could be interpreted as each feature decaying at the same rate and as a probabilistic decay of each feature leading to a uniform rate of forgetting; this question is beyond the scope of the methods used here. Clarifying the nature of random forgetting in VWM remains an important question for future research.

## Conclusions

In this study, we examined the weighting of facial features when storing facial identity. We employed a novel face-morphing procedure, where each facial feature could be separately manipulated in a change detection task. With psychophysical reverse correlation, we were able to estimate internal memory weights for each facial feature, revealing the extent to which the feature was used in the VWM task. We found that eyes were the dominant feature that drives face identity VWM. The mouth, eyebrows, forehead, nose, and cheeks had positive but small weights. The features found corresponded to previous research on face perception, suggesting that VWM representations of faces retain many properties of perceptual representations. As the overall weightings for human participants were surprisingly similar to an optimal stimulus information classifier, which also had large weighting for a subset of facial features, we conclude that human VWM representations are rather efficient in encoding facial information, and internal noise, instead of inefficient representation, may limit the VWM representation. We found little change in facial feature weighting between retention times, but internal noise increased with longer delays. Conversely, sampling efficiency did not decrease with retention time. These results support a straightforward temporal decay model, with a time-dependent increase in internal noise affecting all facial features similarly, rather than some features decaying before others. We conclude that in VWM representations of faces, a few features, mostly the eyes, are weighted heavily; that humans use facial information efficiently in storing facial identities in VWM; and that forgetting in face VWM is likely mediated by random corruption of representations caused by accumulation of internal noise.

## Supplementary Material

Supplement 1
