# Supplementary material for: Facial feature representations in visual working memory: A reverse correlation study
Source: J Vis. 2025 Oct 24;25(12):23. doi: 10.1167/jov.25.12.23 (PMC12574738; doi:10.1167/jov.25.12.23)
Supplement: Supplement 1 [file jovi-25-12-23_s001.pdf]

Supplementary material

Appendix I – Facial morphing

Figure 6 shows the 126 morph points and their groupings that were used in generating stimuli. There were 140 points in total, including 10 points tracking the outlines of the ears and the neck to smooth the edges of the faces, as well as 4 points in each corner of the image generated by the morph algorithm (these points are not shown here). The morph points were grouped in seven facial features: forehead, eyes, eyebrows, cheeks, jaw, nose and mouth (shown in Figure 6 in different colors).

Figure A1. The colored dots represent the morph points used to generate the facial morphs. The morph groups are denoted by different colors. The figure shows one example image, but the same process of assigning points was done for each photograph in equivalent physical locations of the face (e.g., the center of the nose tip). Morph points were added before any image processing to colored photographs of individuals; the figure shows a grayscale photograph for clarity.

Appendix II – individual memory weights

Figure 7 shows normalized memory weights for individual participants calculated over all their experiment trials. The weightings show idiosyncrasies, but most of the participants share similar feature weightings, with the eyes carrying the largest memory weights. In the main analysis, these weights were averaged to grand average weightings.

Figure A2. Normalized memory weights for individual participants. Error bars represent *SEM*.

Figure A1

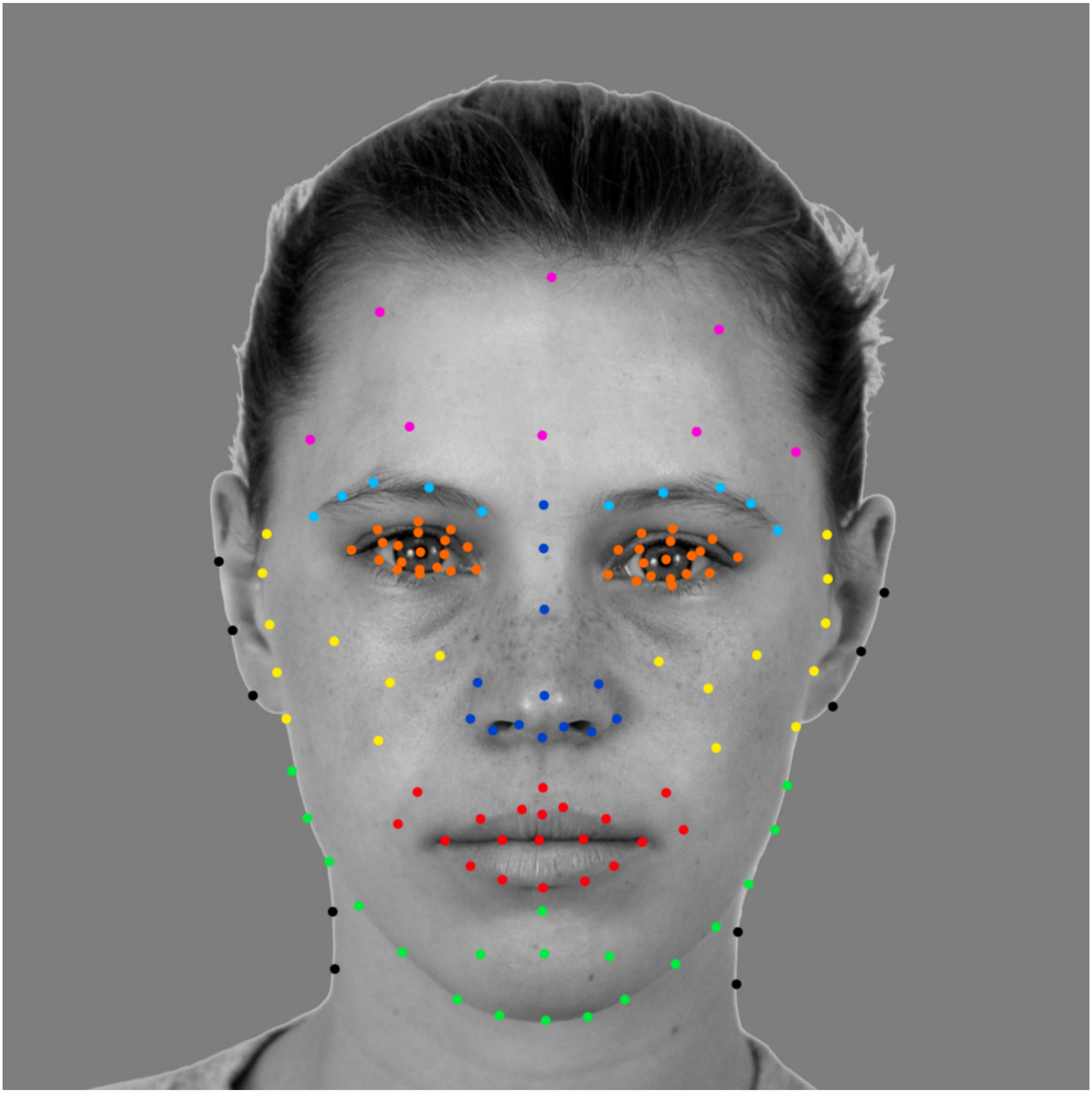

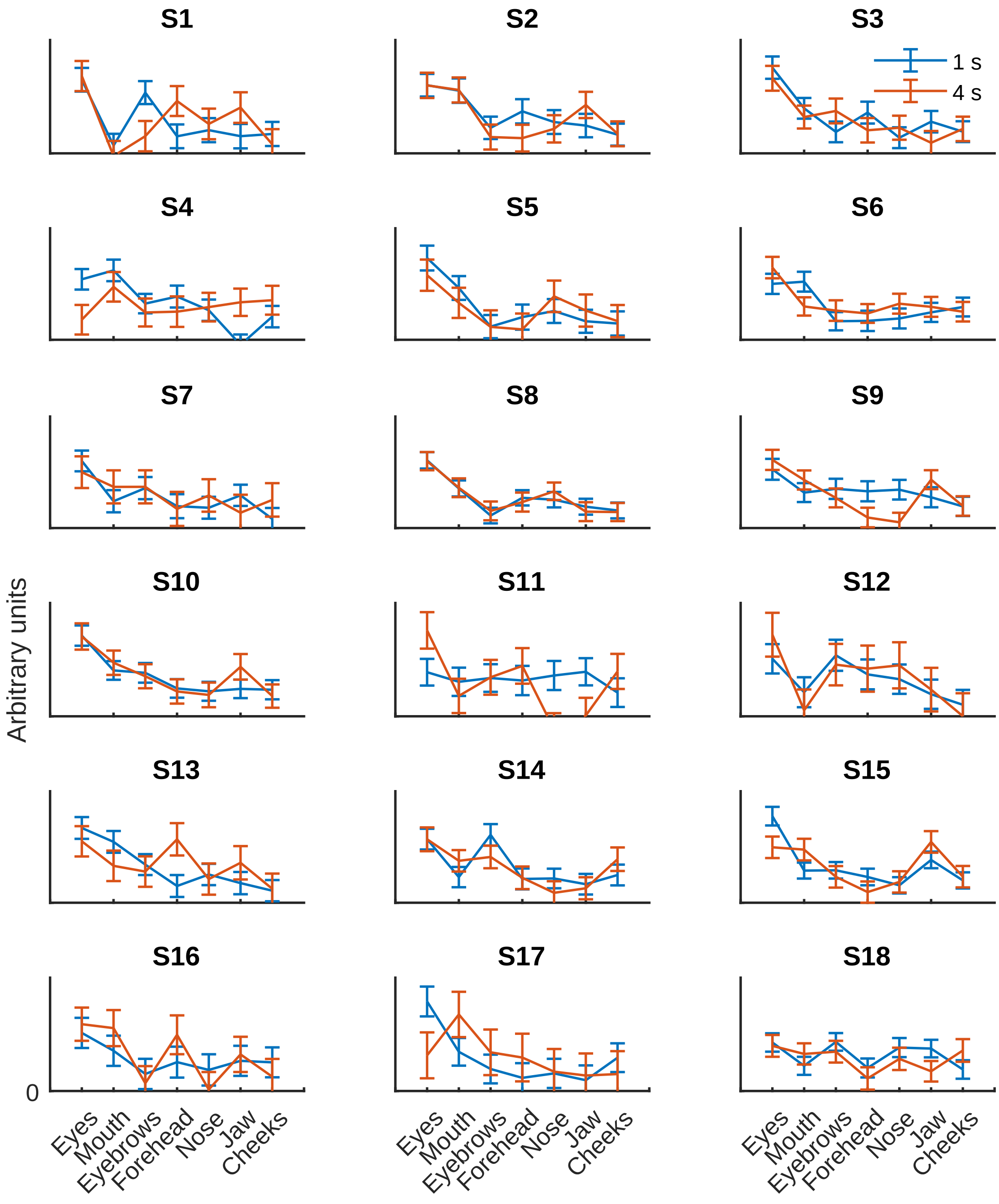
S1
S2
S3
1 s
4 s
S4
S5
S6
S7
S8
S9
S10
S11
S12
S13
S14
S15
S16
S17
S18
Arbitrary units
0
Eyes
Mouth
Eyebrows
Forehead
Nose
Jaw
Cheeks
